# New advances in leukaemia immunotherapy by the use of Chimeric Artificial Antigen Receptors (CARs): state of the art and perspectives for the near future

**DOI:** 10.1186/1824-7288-37-46

**Published:** 2011-09-22

**Authors:** Ettore Biagi, Virna Marin, Greta Maria Paola Giordano Attianese, Irene Pizzitola, Sarah Tettamanti, Elisabetta Cribioli, Andrea Biondi

**Affiliations:** 1 Centro di Ricerca Fondazione "Matilde Tettamanti", Clinica Pediatrica Azienda Ospedaliera San Gerardo, Università Milano-Bicocca, via Pergolesi 33, Monza, 20900, Italy

**Keywords:** Leukaemia immunotherapy, cell therapy, gene therapy, chimeric artificial receptors

## Abstract

Leukaemia immunotherapy represents a fascinating and promising field of translational research, particularly as an integrative approach of bone marrow transplantation. Adoptive immunotherapy by the use of donor-derived expanded leukaemia-specific T cells has showed some kind of clinical response, but the major advance is nowadays represented by gene manipulation of donor immune cells, so that they acquire strict specificity towards the tumour target and potent lytic activity, followed by significant proliferation, increased survival and possibly anti-tumour memory state. This is achieved by gene insertion of Chimeric T-cell Antigen Receptors (CARs), which are artificial molecules containing antibody-derived fragments (to bind the specific target), joined with potent signalling T-Cell Receptor (TCR)-derived domains that activate the manipulated cells. This review will discuss the main application of this approach particularly focusing on the paediatric setting, raising advantages and disadvantages and discussing relevant perspectives of use in the nearest future.

## Introduction

**Figure 1:**
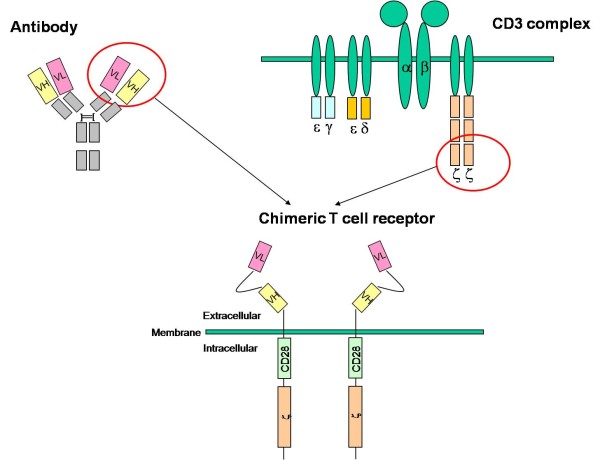
**the structure of a CAR**.

Chimeric T-cell Antigen Receptors (CARs) are a fascinating bio-technologic step in the field of immunotherapy to orient the activity of immune cells towards specific molecular targets expressed on the cell surface of various tumours, including haematological malignancies. CARs are artificial T-cell receptors constituted by an antigen-recognizing extracellular domain derived from an antibody molecule linked to a T-cell triggering domain [1-4]. CARs are generated by joining the heavy and light chain variable regions of a monoclonal antibody, expressed as a single-chain Fragment variable (scFv) molecule, to an intracellular signalling domain, usually the zeta-chain of the TCR/CD3 complex or the gamma-chain from the Fc-epsilon-RI receptor Figure 1. T lymphocytes genetically engineered to express CARs exhibit specific lysis towards tumour cells and cytokine secretion upon exposure to the respective target antigen. The CAR-mediated effector function may produce sustained tumour cell lysis more likely than humoral immune responses alone, based on the use of monoclonal antibodies. The perforin/granzyme killing mechanism may be effective against cells that are relatively resistant to antibody and complement, while cytokine secretion recruits additional components of the immune system, amplifying and prolonging the anti-tumour immune response. Moreover, effector T cells display efficient tumour penetration and homing capabilities [1-4].

The CAR approach permits to overcome the major limitations associated with the use of a "classical" TCR transgenic molecule. In fact, target recognition by CAR is non-MHC restricted and independent of antigen processing, therefore allowing its use in patients with different haplotypes and bypassing tumour escape due to MHC-molecules down-regulation. In addition, CARs can be targeted toward molecules other than peptides, like carbohydrates and glycolipids, and there is no risk to trigger unpredictable and potentially harmful specificities, as it may happen with transduced TCR, that could form hybrids with the endogenous TCR [1-4].

Different CARs have been generated so far [2,3,5], against a wide range of surface molecules expressed by many solid tumours and haematological malignancies. The efficacy of this strategy has been recently proved by a phase I clinical trial in patients with neuroblastoma. In this study, the authors generated a CAR directed to the diasialoganglioside G(D2a), a tumour-associated antigen expressed by human neuroblastoma cells. They demonstrated that Epstein-Barr virus (EBV)-specific cytotoxic T lymphocytes (CTLs) engineered to express the G(D2a) specific-CAR survive longer than T cells activated by the CD3-specific antibody OKT3 expressing the same CAR, but lacking virus specificity. Moreover, infusion of these genetically modified cells was associated with tumour regression or necrosis in half of the subjects tested [6].

## How to build a Chimeric Artificial Receptor

**Figure 2:**
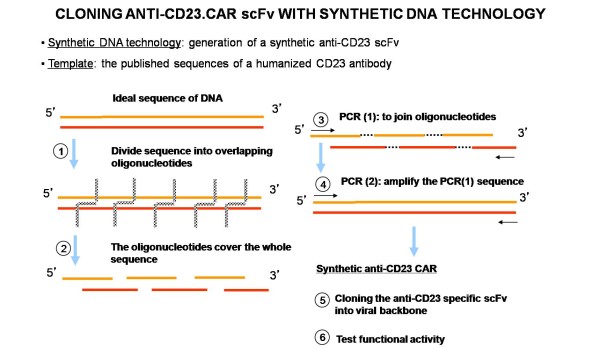
**the Synthetic DNA Technology applied to the anti-CD23.CAR**.

The ectodomain represents the extracellular part of the artificial T-cell receptor. It is generally composed by a signal peptide, an antigen recognition region and a spacer sequence. The signal peptide directs the nascent protein into the endoplasmic reticulum. This is essential if the receptor needs to be glycosylated and anchored in the cell membrane leading to the CAR expression. The antigen recognition domain is usually a scFv, obtained by the fusion of the variable regions of the heavy (VH) and light (VL) chains of Immunoglobulins (Igs), joined together with a short linker. The latter is usually a serine-glycine rich motif, whose flexibility assures a high mobility capacity to the molecule Figure 1. This chimeric domain retains the specificity of the original Ig, despite removal of the constant regions and the introduction of a linker peptide. The traditional method to obtain the scFv was based on the use of mouse hybridoma producing the specific antibody necessary to have the variable fragments of the VL and VH chains [7]. A new approach, more suitable in this contest, is represented by the technology of synthetic DNA. To generate the scFv binding domain by a "synthetic DNA " approach, guidelines published by Rydzanicz et al. can be adopted [8]. As a DNA template for the VH and VL fragment it is possible to use a humanized antibody published sequence. Once the final scFv sequence containing the leader sequence and the flexible linker is found and projected, it is possible to proceed with the synthetic DNA generation. The Rydzanicz et al. method is based on dividing the putative sequence into different overlapping oligonucleotides. To generate specific and functional overlapping oligonucleotides, they created a free online software "Assembly PCR oligo maker" http://www.ncbi.nlm.nih.gov/pmc/articles/PMC1160141/. This software calculates the overlapping oligonucleotides following the putative sequence with specific values of T melting (Tm), thus avoiding non-specific and random annealing possibilities. The overlapping oligonucleotides are then joined together exploiting a basic PCR reaction in order to generate the putative template. A second reaction of PCR is then needed to amplify the new synthetic template using flanking primers Figure 2.

## Clinical application in haematology

Haematological malignancies represent optimal targets for the exploitation of CARs, because of the bright expression of specific antigens on the surface of tumour cells. Previous studies from our group demonstrated that B-lineage acute lymphoblastic leukaemia can be efficiently targeted by a CAR directed against the CD19 antigen [9,10]. We have shown that, after transduction with anti-CD19 CARs, cytokine induced killer (CIK) cells, a peculiar population of effector cells, acquire potent anti-leukemic activity in terms of cytotoxicity, proliferation and release of immunostimulatory cytokines upon specific antigen recognition. In line with these observations, an European consortium http://www.childhope.eu/contents.php have been developed, funded in the contest of a STREP (6^th ^framework) to target high-risk B-ALL in molecular relapse after transplantation using donor-derived anti-CD19. CAR-expressing EBV-CTLs. The preclinical *in vivo *data, targeting primary patient-derived high-risk B-ALL [11], demonstrated the efficacy of such an approach and paved the way, for the first time in the field of paediatric leukaemia, for the design of a phase I clinical trial. In this trial paediatric patients, who received allogeneic transplantation, will be infused with donor-derived anti-CD19. CAR-expressing EBV-CTLs, as soon as the reappearance of the disease is detected by early Minimal Residual Disease (MRD) analysis. A single injection will be performed after a lympho-depletion schedule by fludarabine and discontinuation of imunosuppression.

Similarly, four other main groups are developing CAR-based strategies for targeting B-origin leukaemia-lymphoma neoplasm, by using CD19 as the selected target. Indeed, the MK Brenner's group has recently published interesting even though preliminary observations concerning the *in vivo *superior effect of second generation CARs containing, beyond the zeta-chain, a costimulatory molecule, specifically the CD28 domain. Patients with B cell lymphomas were infused with two different autologous T-cell products expressing CARs with the same specificity for the CD19 antigen: one CAR encoded both the costimulatory CD28 and the zeta endodomains, while the other presented the zeta endodomain alone. CAR-expressing T cells containing the CD28 endodomain showed markedly enhanced growth and persistence compared with CAR-expressing T cells encoding the zeta endodomain alone [12]. Similar and promising results have been obtained in humans by the CH June's and SA Rosenberg's groups, targeting the CD19 antigen in lymphoma patients by using gene-modified autologous T cells [13,14]. Both groups observed and measured long-lasting eradication even in case of disseminated tumours, either by single or multiple injections of anti-CD19. CAR-expressing T cells. Lastly, the LJ Cooper's group has recently proposed a clinical-grade non-viral method to transduce T cells with the anti-CD19. CAR: the "Sleeping Beauty" transposon/transposase system. This innovative method is based on the nucleofection/electroporation of plasmidic DNA, allowing cells to be stably transduced in their nucleus and avoiding the use of viral vectors [15].

Other potential optimal targets of CAR-gene transfer are represented by Acute Myeloid Leukaemia (AML) and Chronic Lymphocytic Leukaemia (B-CLL), because of the selective expression of tumour-associated surface antigens, possible targets of CAR recognition.

**Figure 3:**
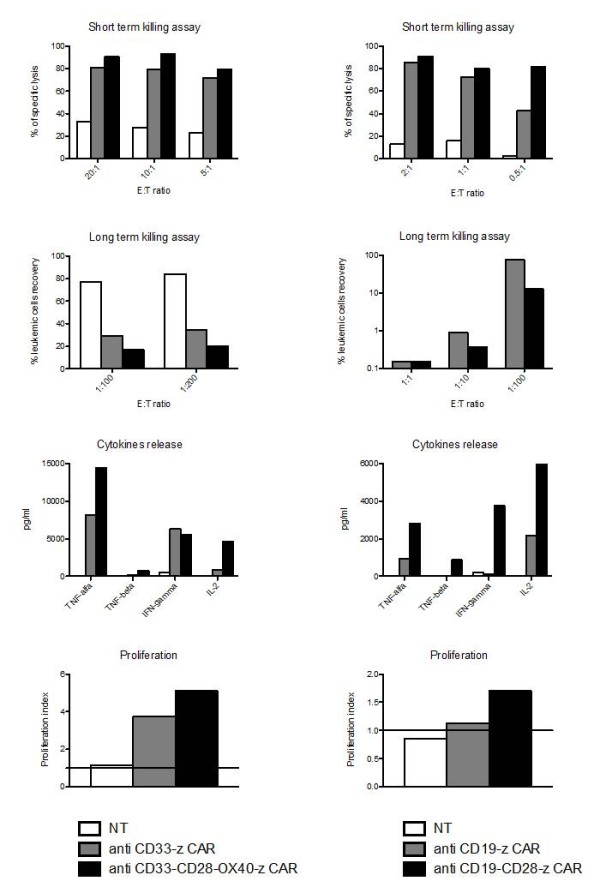
**CARs activity towards myeloid (CD33^+^) and lymphoid (CD19^+^) acute leukaemias**. The figures show different tests adopted to measure the efficacy of transduced T cells. The panels on the left side describe the effect on AML cells, whilst the right panels on ALL cells. The test shown are, starting from the top: 1) short term cytotoxicity, by classic Chromium release assay; 2) long-term cytotoxicity, measured as recovery of leukaemia cells after 1-week of co-incubation with effectors cells; 3) cytokine release, by Elisa tests; 4) proliferation index, measured as ration versus the basal number by thymidine incorporation assay. All the tests are described in details in reference no. 19.

Immunotoxin-based therapy against CD33^+ ^myeloid leukaemia using gemtuzumab ozogamicin (Mylotarg) [16] had some success, albeit associated with liver (Veno-Occlusive Disease) and haematological toxicity in a significant number of patients [16]. With the exception of acute promyelocytic leukaemia, the curative potential of Mylotarg as a single agent is still limited [16]. Immunotherapy with T cells using unmanipulated donor lymphocyte infusion (DLI) for the treatment of leukaemia recurrence in Haematological Stem Cells Transplantation (HSCT) recipients has shown some positive results in AML, but the use of DLI carries a significant risk of inducing Graft versus Host Disease (GvHD) [17]. The identification of leukaemia-associated antigens eliciting T-cell responses *in vitro*, has been successful for AML [18]. However, the generation of leukaemia-specific cytotoxic T cells against AML-associated antigens has been hampered by several factors: the poor immunogenicity of blasts lacking costimulatory molecules and the low frequency of T-cell precursors in antigen-naïve marrow donors or in patients anergized by their own leukaemia. An attractive strategy to overcome these limitations may be represented by the use of human T cells that bear CARs specific for CD33 molecule. In line with these considerations, we have recently published an *in vitro *model that strongly demonstrates the efficiency of anti-CD33. CARs towards myeloid cell lines and primary blasts, by using gene modified CIK cells [19]. The infusion of CIK cells in patients with AML relapsing after HSCT is well tolerated, but limited clinical responses were observed [20]. To improve their effector functions against AML, CIK cells were genetically modified with CARs specific for the CD33 myeloid antigen by using SFG-retroviral vectors encoding for anti-CD33-zeta and anti-CD33-CD28-OX40-zeta chimeric receptors. Anti-CD33. CARs modified CIK cells acquired potent cytotoxicity (up to 80% lysis) against various AML targets, also confirmed in long-term killing experiments. Moreover, a relevant CD33-specific proliferation was measured, accompanied by the release of high levels of immunostimulatory cytokines. The presence of CD28-OX40 in CAR endodomain was associated with a significant amelioration of the anti-leukaemia activity of modified CIK cells. Representative data showing anti-CD33 and anti CD19 CAR-mediated killing and immunostimolatory capacities are shown in Figure 3. Importantly, even though a certain toxicity against normal haematopoietic CD34^+ ^progenitor cells was measured, their residual haematopoietic activity was anyway preserved [19], thus opening the way for possible clinical applications of the anti-CD33. CAR strategy. *In vivo *experiments are currently ongoing in order to measure the safety and efficacy profile of anti-CD33. CAR-expressing cells in an immunodeficient mouse model.

Similarly to ALL and AML, B-CLL may represent as well an interesting target for CAR strategy. B-CLL cells expressing CD19 and CD20dim are suitable to be targeted with T cells redirected against these pan-B markers. In fact, CARs have been generated to target these self tissue-restricted B-cell antigens [12-15]. However, B-CLL hypo-gammaglobulinemia is present in approximately 8% of patients at diagnosis and progressively increases along the natural history of the disease. Thus, targeting pan-B markers can potentially further compromised the immunity of these patients. A more selective self-antigen in B-CLL that can be more suitable is the CD23 molecule, because of its higher expression on B-CLL cells as compared to normal B lymphocytes [21]. CD23 is the low-affinity receptor for IgE and its expression and function is physiologically restricted to IgE-secreting B lymphocytes, mast cells, platelets and dendritic cells. Lumiliximab (L-mab), a macaque-human chimeric anti-CD23 monoclonal antibody, has been used in a Phase I-II study, to test its safety, efficacy, and pharmacokinetics. In the last study 31 patients received either 375 mg/m(2) (n = 3) or 500 mg/m(2) (n = 28) of L-mab in combination with Fludarabine, Cyclophosphamide and Rituximab (FCR) for 6 cycles [22]. The overall response rate was 65%, with 52% of patients achieving a complete response (CR). Study-related adverse events were mild and evidence of clinical activity consisted of reductions in absolute lymphocyte counts and lymphonodes size. T cells redirected against CD23 through a specific CAR may further improve these results. In line with these observations, we have recently published an *in vitro *and *in vivo *model that corroborates this hypothesis [23]. We cloned and expressed in T lymphocytes a novel CAR targeting the CD23 antigen. Anti-CD23. CAR-expressing T cells showed specific cytotoxic activity against CD23^+ ^tumour cell lines (average lysis 42%) and primary CD23^+ ^CLL cells (average lysis 58%). This effect was obtained without a significant toxicity against normal B lymphocytes, in contrast to CARs targeting CD19 or CD20 antigens, which are also physiologically expressed by normal B lymphocytes. Moreover, autologous CLL-derived-CD23. CAR-expressing T cells significantly released inflammatory cytokines and IL-2 was also produced to sustain the CD23-driven proliferation of such modified T cells. Redirected T cells were also effective *in vivo *in a CLL Rag2(-/-)γc(-/-) xenograft mouse model [24]. Indeed, the infusion of anti-CD23. CAR-expressing T cells resulted in a significant delay in the growth of the CLL-derived cell line (MEC-1). These data suggest that anti-CD23. CAR-expressing T cells represent a selective immunotherapy approach for the elimination of CD23^+ ^leukaemia cells in patients affected by B-CLL.

Despite the promising results obtained so far, CAR strategy might be further optimized. Several reports indicate that the addition of costimulatory molecules (such as CD28, ICOS, CD134 or CD137), either as single domain or as multiple combinations, to the signalling CAR endodomain induces a higher rate of proliferation, higher levels of IL-2 secretion and prolonged effector cells survival with a subsequent better activity *in vivo *[25]. An additional approach to ameliorate CAR-mediated effector functions might be the inactivation of inhibitory molecules. These inhibitory ligands are expressed on various tumour cells and are associated with functional impairment of extensively *in vitro *stimulated T cells: their blockade can accelerate tumour eradication [26]. A different strategy to guarantee an improved and totally physiological activation of T cells can be achieved exploiting an activated native TCR, such as a viral targeting TCR, in addition to the CAR specificity (the so-called "dual-specific" T cells) [6]. In this condition, the activation of the natural TCR should provide a powerful stimulus, maintaining at the same time the killing capacity, the proliferative activity and the durable persistence of the CAR-related functions. Indeed, MK Brenner's group has recently adopted EBV-specific CTLs, transducing them with a CAR targeting the G(D2a) antigen expressed by neuroblastoma cells. Such manipulated cells preserved an intact activity towards the viral target and also killed the neuroblastoma cells through the anti-G(D2a). CAR. As described above, this strategy has been proved to be feasible and efficient in a recent phase I clinical trial [6].

Alternatively to the use of EBV-CTLs, other T-cell subsets have shown some potential for future adoptive immunotherapeutic approaches. In line with previous considerations, there is enough evidence to support the *in vivo *use of chimeric EBV-CTLs in paediatric relapsed/refractory ALL patients, as planned in a Phase I trial in the context of the "Childhope" STREP-funded network. However, additional preclinical experiments are crucial to compare the respective anti-tumour efficacy of chimeric EBV-CTLs, chimeric CIK cells and chimeric gamma9delta2 T cells (GDT) for future clinical applications. CIK cells are T cells with natural killer cell phenotype and function, enriched in CD3+CD56+ cells [19], that can be easily expanded from human peripheral blood mononuclear cells up to 200-1000 fold in 14-21 days of culture after an initial priming with IFN-gamma and OKT-3 followed by repeated stimulation with high-doses of IL-2 [20]. It has been demonstrated that CIK cells can lyse a broad array of tumour targets in a non-MHC-restricted manner and display an *in vivo *potent anti-tumour activity, while having reduced propensity to induce GvHD [27]. Phase I studies have been performed with CIK cells in various contexts, where no toxicity was observed and several partial responses were detected [20-27]. Similarly, human peripheral GDT T cells are a proved alternative to conventional CTLs. The vast majority of peripheral blood GDT cells, that represent 0.5 to 5.0% of whole peripheral blood T cells in humans, express TCR composed of a restricted set of variable regions called Vgamma9 and Vdelta2. Beside their known anti-infectious activity -in particular following allogeneic HSCT- it was shown that GDT cells are able to kill a wide variety of tumour cell lines from very diverse origins [28]. GDT cells can be selectively expanded *in vivo *or *in vitro *in the presence of aminobisphosphonates without prior antigen priming [29]. Recently, it has been shown that human GDT cells can be expanded in the presence of zoledronate and efficiently transduced with a retroviral vector encoding a CD19-specific CAR. Transduced GDT cells powerfully recognised and lysed antigen-expressing tumour cell targets [30]. To unravel such an important issue, we have recently published a comparison, in an *in vitro *model, of the above mentioned populations as putative effectors of CAR-mediated immunotherapy [31]. Our results indicate that the expression of an anti-CD33-zeta chimeric receptor potently and similarly increases the antileukaemic functions of different effector T-cell subtypes. This underlines the impossibility to identify a more potent T-cell population through an *in vitro *analysis. Moreover, these results are consistent with recent observations that have emerged from clinical trials with CAR-modified T cells, suggesting the need to perform such type of studies in the human setting.

An important consideration for the clinical applicability of CAR strategy is represented by the safety issues concerning the use of integrating vector for gene transfer, as well as the potential reactivity of CAR-transduced cells against host-normal cells expressing the target antigen. Furthermore, the potential hazards associated with the use of CAR containing costimulatory or growth-promoting molecules that may improve the survival and the proliferation of manipulated T cells need to be investigated [1-4]. These limitations can be overcome by introducing suicide genes that constitute a back-up control to be used in case of unexpected reactivity of transduced cells [32]. Several suicide gene strategies have been described so far. The most described approach has been based on the thymidine kinase gene of the Herpes Simplex virus (HSV-TK). Expression of the HSV-TK gene by transduced T cells renders them susceptible to the effects of gancyclovir (GCV). Several groups have reported that transduced T cells retained their anti-leukaemia effect, including long-lasting remissions in some patients [33]. However, HSV-TK is highly immunogenic in humans. The most recently described strategy is the inducible Casp9 suicide system, which is based on a fusion protein constituted by human caspase 9 and the modified human FK506 binding protein (FKBP), that allows conditional dimerisation triggered by a chemical inducer of dimerisation (CID) [34]. Other suicide genes that have been studied include the human CD20, which is targeted by the monoclonal antibody Rituximab [35], and a mutant human thymidylate kinase (mTMPK), which renders transduced cells susceptible to zidovudine (AZT) [36]. A fundamental step to identify the optimal suicide approach for clinical intervention requires a direct comparison of all these systems. Our preliminary results (*unpublished data*) confirm a substantial equivalence of the HSV-TK and Casp9-CID systems, having the latter the double advantage of not being immunogenic and exerting its activity in a more rapid timeframe.

## Conclusions

In conclusion, CARs represent a potential useful tool to fight various resistant forms of leukaemia. As recently discussed by Helen Heslop [37], more than 10 clinical trials using second or third-generation CARs are currently present on http://ClinicalTrials.gov, and there is some encouraging preliminary evidence of their clinical efficacy. On the other side, two serious adverse events have been reported. The first event [38] occurred in a patient with largely metastatic colon cancer who was infused with more than 10^10 ^T cells expressing a third-generation CAR targeting HER2, after being pre-treated with an intensive lymphodepletion regimen. The patient rapidly presented pulmonary toxicity within few minutes, followed by cardiac arrest and finally died few days later. The investigators concluded that the toxicity was due to targeting of low levels of HER2 on pulmonary endothelium by transgenic T cells. In the second clinical case, Brentjens and colleagues described a patient with bulky CLL and extensive previous chemotherapy treatment who received autologous T cells expressing a second generation anti-CD19. CAR at a dose of 3 × 10^7 ^cells/kg after being lymphodepleted with cyclophosphamide [39]. This patient suddenly developed fever, hypotension, and dyspnea few hours after infusion, with rapid fatal progression. An autopsy did not reveal a clear cause of death. It was concluded that low grade sepsis was the most likely triggering factor in this heavily pre-treated patient, but it was also hypothesized that a cyclophosphamide-induced "cytokine storm" may have potently augmented the *in vivo *activation process of modified T cells.

All these aspects have been currently widely considered and discussed by many researchers and regulatory experts in the field of cell and gene therapy. Different schedules of administration (dose-escalation) with a reduced cell numbers or fractionated doses, together with a better choice of pre-administration lympho-depletive regimens, in patients with minimal residual disease, would probably diminish the possibility of observing severe adverse events. Withal, the use of CARs containing suicide-gene switch-off mechanisms could be helpful in case of off-target side events [4,40].

## Competing interests

The authors declare that they have no competing interests.

## Authors' contributions

EB and AB conceived and wrote the article. VM and GMPGA performed and wrote the literature search. VM and EC edited the parts (and performed literature search) concerning applications of CARs in B-cell malignancies. VM, IP and ST edited the parts (and performed literature search) concerning applications of CARs in AML. GMPGA edited the parts (and performed literature search) concerning applications of CARs in CLL. GMPGA, IP, ST, EC contributed to figures preparation. VM edited the article for publication. All authors have read and approved the script.
